# Genome-Wide Identification of the Trihelix Transcription Factor Family and Functional Analysis of *ZmTHX15* in Maize

**DOI:** 10.3390/ijms252413257

**Published:** 2024-12-10

**Authors:** Yanyong Cao, Zeqiang Cheng, Xinyan Sun, Meichen Zhu, Ling Yue, Hui Liu, Xiaolin Wu, Jinghua Zhang, Canxing Duan

**Affiliations:** 1 Institute of Cereal Crops, Henan Academy of Agricultural Sciences, The Shennong Laboratory, Zhengzhou 450002, China; yanyongcao@126.com (Y.C.); zeqiangcheng@163.com (Z.C.); xinyansun123@163.com (X.S.); zmc910824@163.com (M.Z.); 2 National Key Laboratory of Wheat and Maize Crop Science, College of Life Sciences, Henan Agricultural University, Zhengzhou 450046, China; 18838106740@163.com (L.Y.); liuhuisw@henau.edu.cn (H.L.); wuxiaolin@henau.edu.cn (X.W.); 3 Key Laboratory of Grain Crop Genetic Resources Evaluation and Utilization, Institute of Crop Sciences, Chinese Academy of Agricultural Sciences, Beijing 100081, China

**Keywords:** drought stress, gene expression, cis-acting elements, *ZmTHX*

## Abstract

The trihelix transcription factor, which is a plant-specific family, play a critical role in plant growth and development and stress responses. Drought is the main limiting factor affecting yield of maize (*Zea mays*). However, the identification and characterization of this gene family in maize and its biological functions in response to drought stress have not been reported. Here, 46 *Zea mays* trihelix genes (*ZmTHXs*) were identified in the genome. Phylogenetic analysis of the *ZmTHXs* revealed that the genes were clustered into five subfamilies: GT-1, GT-2, GTγ, SH4, and SIP1. Chromosomal localization analysis showed that the 46 *ZmTHXs* were unevenly distributed across 10 chromosomes in maize. Cis-acting elements related to abiotic stress in *ZmTHXs* were found. Most *ZmTHXs* genes showed significant changes in expression levels under drought treatment. In addition, *ZmTHX15*-overexpressing Arabidopsis exhibited stronger drought tolerance with less secondary oxidative damage and higher photosynthetic rate. These findings could serve as a basis for future studies on the roles of *ZmTHXs* and the potential genetic markers for breeding stress-resistant and high-yielding maize varieties.

## 1. Introduction

The growth and development of plants and their adaptation to the environment are regulated by multiple genes. Transcription factors, the most important regulatory elements, are widely involved. Also known as trans-acting factors, transcription factors can bind to specific cis-acting elements of eukaryotic genes to regulate the transcription of related genes, thereby enabling the target genes to express in a specific temporal and spatial manner [1]. To date, more than 64 types of transcription factors have been identified in higher plants, playing crucial roles in plant growth, development, and responses to abiotic stresses [2].

Trihelix (THXs) are a unique class of transcription factors specific to plants, first discovered and isolated in peas [3], and play important roles in developmental processes. THXs generally bind to the core sequence 5′-G-Pu-(T/A)-A-(T/A)-3′ in the promoter region of the ribulose-1,5-biphosphate carboxylase small subunit 3A (rbcS-3A) gene, regulating light response reactions [4].

THXs contain three tandem α-helix domains (helix-loop-helix-loop-helix), which is why members of this family are called trihelix transcription factors. THXs can specifically bind to GT elements related to light response to regulate the expression of related genes; thus, they are also known as GT factors [5]. GT elements are highly degenerate cis-acting elements, with the core sequence 5′-GTGTGGTTAATATG-3′ [4].

Research has found that the conserved domains of trihelix transcription factors overlap and share similarities with the helix-loop-helix structure of MYB transcription factors [6]. Therefore, the domains of trihelix transcription factors include the main features of MYB transcription factors [6]. Based on their domains and other sequence characteristics, this family is divided into five subfamilies: GT-1, GT-2, GTγ, SH4, and SIP1 [7]. The GT-2 subfamily has two DNA-binding domains, while the other four subfamilies have only one DNA-binding domain [7]. Each subfamily is named after the first member discovered.

Additionally, based on the recognized elements, members of this family can be divided into three categories: GT-1, GT-2, and GT-3. GT-1 and GT-3 can specifically bind to the Box II (5′-GTGTGGTTAATATG-3′) and 5′-GTTAC-3′ motifs. However, GT-2 is different; its N-terminal can bind to the GT-3 box (5′-GAGGTAAATCCGCGA-3′), while its C-terminal can bind to the GT-2 box (5′-GCGGTAATTAA-3′) [8].

Recent studies have shown that THXs not only respond to light induction but also play important roles in flower organ morphogenesis, trichome formation, embryo development, and grain development [9,10,11,12,13]. For example, the trihelix transcription factor GT-2-LIKE1 (GTL1) and its homolog DF1 inhibit root hair growth by directly binding to the promoter of *ROOT HAIR DEFECTIVE 6-LIKE4* (*RSL4*) in Arabidopsis [14]. In Arabidopsis, the trihelix transcription factor PETAL LOSS (PTL) is involved in the morphogenesis of various floral organs, including the perianth, petal, and stamen [15]. The GT-1 subfamily gene *At5g63420*, which encodes a metal-β-lactamase-trihelix complex, is highly expressed in seeds and is essential for early embryogenesis [16]. Through map-based cloning, a trihelix gene named *Shattering 1* (*SHA1*) was identified in rice that played an important role in regulating seed shattering [17].

In addition to their involvement in plant growth, development, and light responses, THXs also play a crucial role in responding to biotic and abiotic stresses. In Arabidopsis, the trihelix transcription factor *ARABIDOPSIS SH4-RELATED3* (*ASR3*) played a negative role in regulating pattern-triggered immunity (PTI) since the *ASR3* mutant showed enhanced disease resistance to virulent bacterial pathogen infection [18,19]. Another trihelix family member, *ARS3-Interacting Transcriptional Factor 1* (*AITF1*), regulates plant immune responses and resistance to phytobacteria [20]. Overexpression of *AITF1* causes autoimmunity in plants dependently on *ASR3* [20]. A cotton (*Gossypium hirsutum* L.) trihelix family gene, *GhGT-3b_A04*, was strongly induced by the fungal pathogen *Verticillium dahlia* [21]. Overexpression of *GhGT-3b_A04* in Arabidopsis enhanced the plant’s resistance to *Verticillium wilt* [21]. *Arabidopsis thaliana GT-2-LIKE 1* (*AtGTL1*) acts as a positive regulator of PTI since *Atgtl1* mutants are compromised in basal resistance to *Pst* DC3000 infection, whereas the overexpression of *AtGTL1* leads to a reduced susceptibility [22]. In kiwifruit, the trihelix transcription factor GT1 interacts with E3 ubiquitin ligase PUB23 to negatively regulate immune responses against *Pseudomonas syringae pv. actinidiae* [23]. *ZmGT-3b*, a GT-1 subfamily trihelix transcription factor, negatively regulates plant defense responses as knocking down of *ZmGT-3b* in seedlings led to enhanced resistance to *Fusarium graminearum* infection [24].

A study has found that *AtGT-4* interacted with AP2/ERF domain-containing protein (TEM2) to positively co-regulate the expression of the salt responsive gene *Cor15A*, thereby enhancing salt stress tolerance in Arabidopsis [25]. The transcript level of *OsGTγ-1* is strongly induced by salt stress, and mildly induced by drought, low temperature, and ABA treatment [5]. Overexpression of *OsGTγ-1* in rice enhanced the salt tolerance of transgenic plants, whereas the homozygous mutant *osgtγ-1* (T-DNA insertion in the promoter region of *OsGTγ-1*) exhibited reduced salt tolerance and increased sensitivity to salt stress [5]. Moreover, a study has found that *OsGTγ-2* directly bound to the promoter of ion transporting genes to regulate salt response [26]. Overexpression of *OsGTγ-2* in rice improved the seed-germination rate, seedling growth, and survival rate under salt stress. However, CRISPR/Cas9-mediated *OsGTγ-2* knockout lines showed salt-hypersensitive phenotypes [26]. *AtGT2L* was involved in plant responses to low temperature and salt stress, as in plants overexpressing *AtGT2L*, both basal and chilling/NaCl-induced expression levels of cold- and salt-inducible marker genes *RD29A* and *ERD10* were significantly higher than those in wild-type (WT) plants [27]. A member of the cotton trihelix transcription factor (*GhGT26*) positively regulates salt stress as overexpression of *GhGT26* in Arabidopsis improved the tolerance to salt stress [28].

*Arabidopsis SIP1 clade Trihelix 1 (AtAST1)* could regulate the expression of peroxidase (POD), superoxide dismutase (SOD), and late embryogenesis abundant (LEA) genes by binding to the AGAG-box, thus improving plant tolerance to salt and osmotic stress [29]. Transgenic Arabidopsis plants overexpressing *AtAST1* increased the tolerance to drought, salt, and osmotic stress, whereas the *AST1* mutants displayed sensitive phenotype [29]. Chromatin immunoprecipitation (ChIP) analysis indicated that *AtGTL1* interacted with the GT-3 box in the *STOMATAL DENSITY AND DISTRIBUTION1* (*AtSDD1*) promoter, negatively regulating *SDD1* to modulate stomatal density [30]. The expression level of *AtGTL1* was downregulated under water stress, and *GTL1* loss-of-function mutants improved drought tolerance by reducing stomatal density, decreasing water loss through transpiration, and increasing water use efficiency [30]. *TaGT2L1D*, a transcriptional repressor, has similar effects to *AtGTL1* in regulating drought resistance and stomatal aperture [31]. It could suppress the expression of *AtSDD1* by directly binding to the GT-3 box in its promoter that negatively regulated drought resistance [31]. Overexpression of *TaGT2L1D* significantly increased stomatal density and reduced drought tolerance in *GTL1-3* plants [31]. Conversely, plants overexpressing *AtSDD1* gene exhibited reduced stomatal density, decreased transpiration, and improved drought tolerance [31]. Similarly, the *PtaGTL1* gene in poplar (*Populus* L.) enhanced the drought resistance of transgenic plants by regulating leaf stomatal density [32].

A member of the cotton trihelix transcription factors family (*GhGT23*) positively regulates salt and drought stress responses, since overexpression of *GhGT23* in Arabidopsis improved drought and salt tolerance [33]. Overexpression of *Gh_A05G2067* (*GT-2*) showed enhanced drought tolerance with lower malondialdehyde (MDA), hydrogen peroxide contents and higher oxygen-scavenging enzyme activities in cotton [34]. Knocking down of *ZmGT-3b* led to enhanced drought tolerance in maize [24]. *ShCIGT*, a GT-1 subfamily trihelix transcription factor, mediates cold and drought tolerance in tomato [35]. *SlGT-26* negatively regulates drought and salt stress resistance in tomato since *SlGT-26*-RNAi transgenic plants significantly enhanced drought resistance and salt tolerance [36]. *SlGT-30* functions as a negative regulator of drought resistance as CRISPR-mediated loss of function mutant of *SlGT-30* exhibited improved plant performance under drought stress in tomato [37].

Overall, trihelix transcription factors play a crucial role in regulating plant development and abiotic/biotic stress responses. Although many trihelix genes have been reported, their functions still require further investigation. In this study, we used bioinformatics to identify the members, physicochemical properties, classification, chromosomal localization, cis-acting elements, and differential expression analysis under drought stress of the trihelix family in maize. Moreover, *ZmTHX15*-overexpressing Arabidopsis exhibited greater drought tolerance compared to WT plants, with reduced secondary oxidative damage and enhanced photosynthetic rate. These findings provide a foundation for future research on the roles of *ZmTHXs,* which may be potential genetic markers for breeding stress-resistant and high-yielding maize varieties.

## 2. Results

### 2.1. Identification and Physicochemical Properties of ZmTHXs

To determine the composition and physicochemical properties of the *ZmTHX*s gene family, we identified and analyzed their members and properties. First, searches were conducted in the PlantTFDB and Uniprot databases, using the presence of the MYB binding 4 domain as the criterion for sequence alignment and selection. After removing redundant proteins or those lacking the typical domain, a total of 46 *ZmTHXs* family members were identified. Based on their chromosomal locations, these genes were sequentially named *ZmTHX01* to *ZmTHX46* (Table 1).

Physicochemical property analysis indicated that the 46 members of the *ZmTHXs* family ranged in size from 187 to 863 amino acids (aa), with an average of 421 aa. The molecular weight (MW) of these proteins ranged from 8,855.39 to 96,306.71 kDa. The theoretical isoelectric point (pI) ranged from 4.55 to 11.19, with 22 proteins having a pI above 7, categorizing them as basic proteins, while the remaining proteins had a pI below 7, making them acidic proteins. The instability index, an important indicator for measuring protein stability, ranges from 32.16 to 96.72, with proteins theoretically considered unstable if the index exceeds 40; all family proteins, except for *ZmTHX15* and *ZmTHX35*, are unstable. The average hydropathicity index ranges from −1.259 to −0.296, and proteins with an index below 0 are generally hydrophilic, indicating that all family members are hydrophilic. Subcellular localization predictions showed that, except for *ZmTHX30*, which is localized to the chloroplast, all other members were localized to the nucleus. Although ZmTHXs vary greatly in the number of aa and pI values, they exhibit good similarity in hydrophilicity, instability index, and subcellular localization, indicating a high degree of sequence conservation within this protein family (Table 1).

### 2.2. Phylogenetic Analysis and Subfamily Classification of ZmTHXs

**Figure 1:**
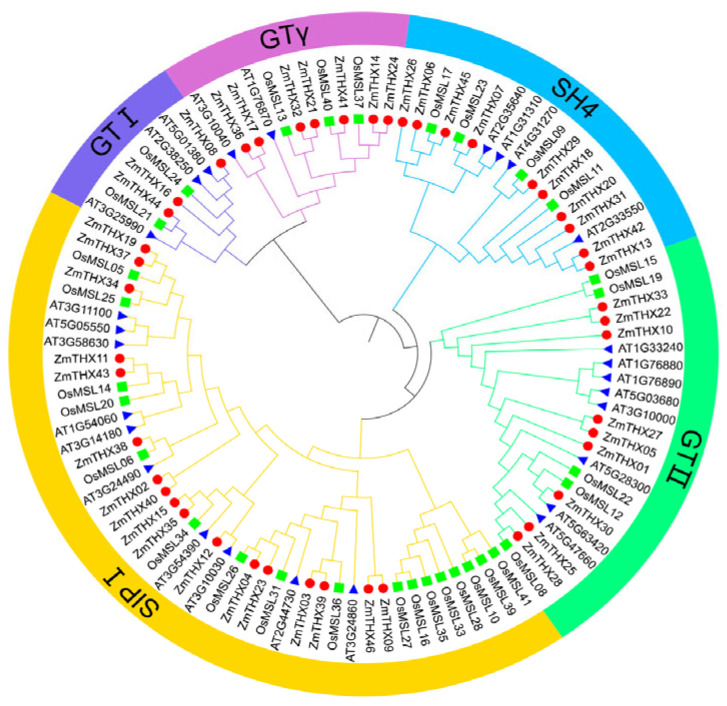
Analysis of the evolutionary relationship of THXs in maize, rice, and *Arabidopsis*.

To better understand the subfamily classification and evolutionary relationships of THX family members, we performed sequence alignment on 27 Arabidopsis THX sequences, 31 rice THX sequences, and 46 maize THX sequences. We used MEGA X and iTOL for the construction and visualization of the phylogenetic tree. The analysis results showed that this family was divided into five subfamilies: GT-1, GT-2, GTγ, SH4, and SIP1 (Figure 1). The SIP1 subfamily had the most members, with 17, accounting for 36.9% of the total family members, while the GT-1 subfamily had the fewest members, with only three, accounting for 6.5% of the family members. The phylogenetic tree showed that the GT-1 and GTγ subfamily members were located on the same branch, indicating that these two subfamilies diverged later and have a closer evolutionary relationship. Each subfamily branch contained THX family proteins from both Arabidopsis and rice. Compared to dicotyledonous Arabidopsis, the THXs of maize and rice generally occupy lower nodes in the branches and converge with Arabidopsis sequences at higher nodes, suggesting a higher degree of homology between maize and rice as they are monocot.

### 2.3. Chromosomal Localization and Synteny Analysis of ZmTHXs

**Figure 2:**
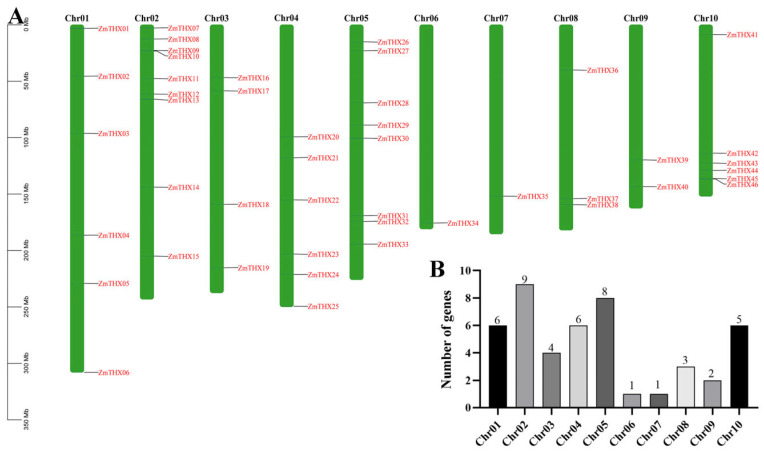
The location of *ZmTHXs* on chromosomes. (**A**) Distribution of *ZmTHXs* on chromosomes; (**B**) The number of *ZmTHXs* on each chromosome.

To determine the distribution of *ZmTHXs* on maize chromosomes, we conducted a chromosomal localization analysis of the family members. The results showed that the 46 *ZmTHXs* are unevenly distributed across 10 chromosomes in maize (Figure 2A). Among them, chromosome 2 had the highest number of *ZmTHX*s, with nine genes, accounting for 19.6% of the total family genes. Chromosomes 6 and 7 had the fewest, with only one gene each. The number of *ZmTHXs* on the other chromosomes ranged from two to eight. The *ZmTHXs* on chromosomes 6 and 7 were located downstream, while the *ZmTHXs* on chromosome 10 were distributed at both ends. The remaining *ZmTHXs* were mostly randomly distributed on various chromosomes (Figure 2B).

**Figure 3:**
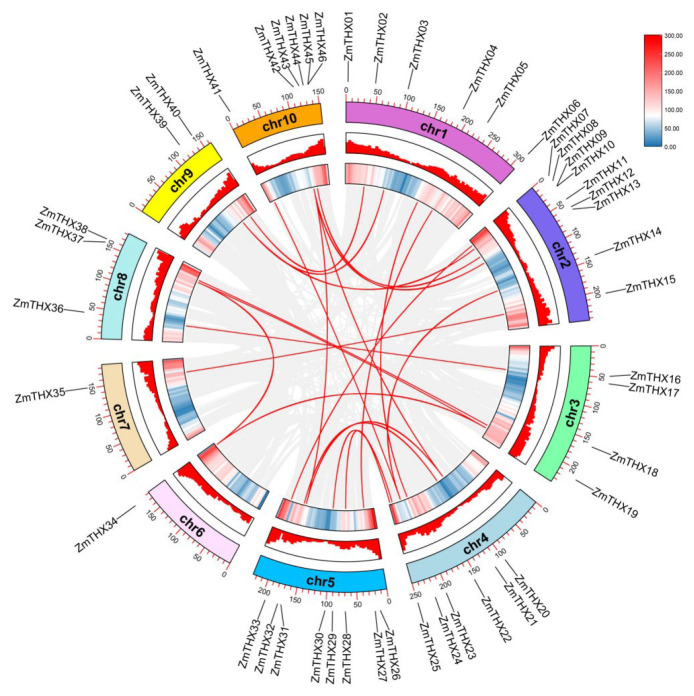
*ZmTHXs* collinearity analysis. Heat maps and histograms along the rectangles represent gene densities on chromosomes. Gray lines indicate syntenic blocks in the poplar genome, and red lines between chromosomes indicate gene pairs with segmental duplications.

Using TBtools, combined with the multiple collinearity scanning toolkit (MCScanX), we analyzed tandem repeat events among *ZmTHXs* genes. The results showed that there are no tandem repeat events within this gene family in maize. By using TBtools in conjunction with BLASTP and MCScanX to analyze segmental duplication events, we identified a total of 23 gene pairs with segmental duplication events. The results indicated that segmental duplication events were a major driving force behind the diversity of *ZmTHXs* genes (Figure 3).

### 2.4. Analysis of Cis-Acting Elements in ZmTHXs

**Figure 4:**
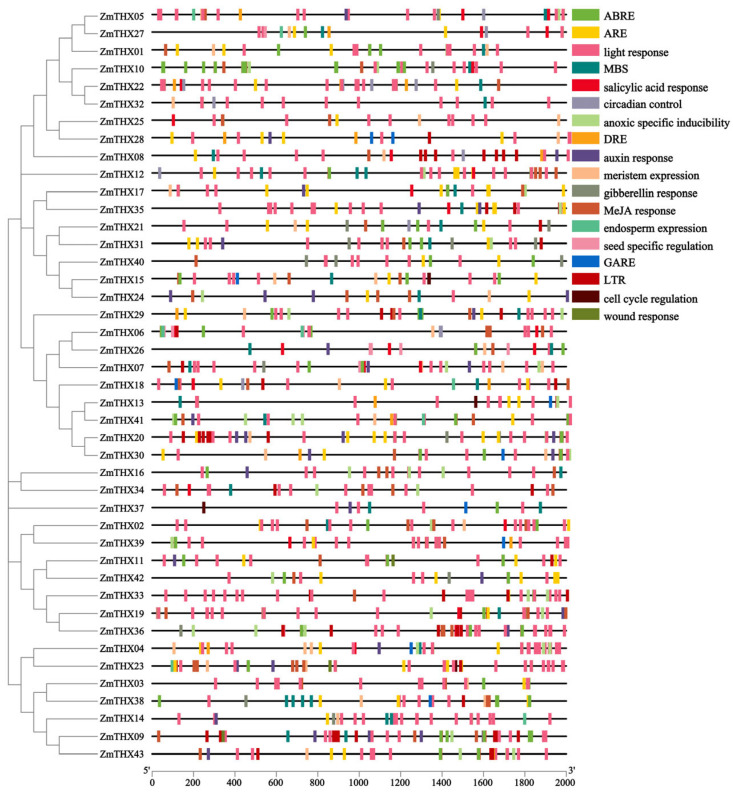
Analysis of cis-acting regulatory elements in *ZmTHXs*. The key cis-acting regulatory elements are distributed in the 2000 bp region upstream of the *ZmTHXs*, and different elements are shown in different colors.

To explore the stress-responsive elements in this gene family, we performed a cis-acting element analysis on the 46 *ZmTHXs* of the family. As shown in the Figure 4, in addition to core enhancer elements, transcription initiation elements, and WRKY binding site elements, 16 types of other elements were identified (Figure 4). All *ZmTHXs* genes contained light-responsive elements and also included five types of phytohormone-related elements: Gibberellins (GA), Auxin (IAA), abscisic acid (ABA), salicylic acid (SA), and Methyl Jasmonate (MeJA) response elements. These cis-acting elements suggested that the expression of ZmTHXs was regulated by various phytohormones. Additionally, we found cis-acting elements related to abiotic stress in *ZmTHXs*, including anaerobic response elements (ARE), hypoxia-specific inducible elements, drought stress response elements (DRE and MBS), and low-temperature response elements (LTR). The results indicated that *ZmTHXs* may play an important role in stress response as well as in plant growth, development, and metabolic regulation.

### 2.5. Gene Expression Profile Analysis

**Figure 5:**
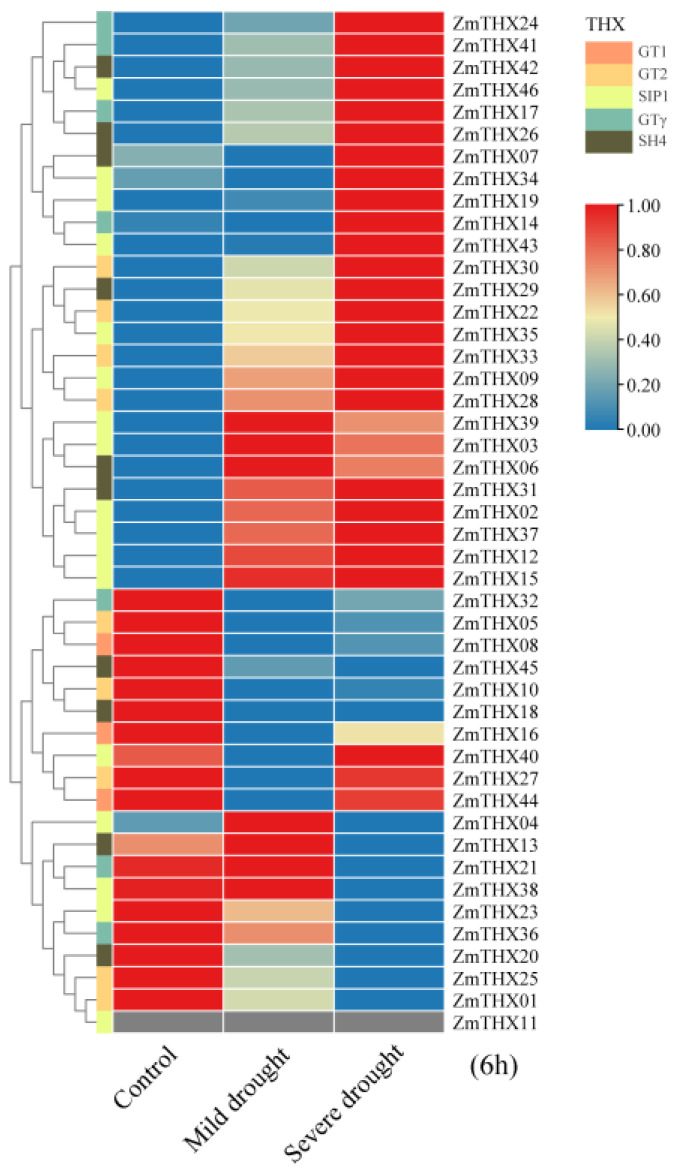
Heat map of differential expression of *ZmTHXs*. Drought treatment time is 6 h. Different-colored rectangles on the right side of the evolutionary tree represent genes of different subfamilies.

To investigate the expression patterns of the *ZmTHXs* under drought stress, we constructed an expression heat map of *ZmTHXs* under drought conditions. The heat map results showed that most genes in this family responded to drought stress. Genes such as *ZmTHX24*, *ZmTHX41*, *ZmTHX42*, *ZmTHX46*, *ZmTHX17*, *ZmTHX26*, *ZmTHX7*, *ZmTHX34*, *ZmTHX19*, *ZmTHX14*, and *ZmTHX43* had low expression levels under normal conditions, showed no significant change in expression level under mild drought (the soil moisture content is between 12% and 16%), but exhibited a significant increase in expression level under severe drought (the soil moisture content is between 6% and 8%) (Figure 5). Genes such as *ZmTHX30*, *ZmTHX29*, *ZmTHX22*, *ZmTHX35*, *ZmTHX33*, *ZmTHX9*, and *ZmTHX28* had increasing expression levels with increasing drought severity (Figure 5). Genes such as *ZmTHX39*, *ZmTHX3*, *ZmTHX6*, *ZmTHX31*, *ZmTHX2*, *ZmTHX37*, *ZmTHX12*, and *ZmTHX15* were relatively sensitive to drought stress and could be highly induced even under mild stress (Figure 5). Genes such as *ZmTHX32*, *ZmTHX5*, *ZmTHX8*, *ZmTHX45*, *ZmTHX10*, *ZmTHX18*, *ZmTHX23*, *ZmTHX36*, *ZmTHX20*, *ZmTHX25*, and *ZmTHX01* had high expression levels under normal conditions but showed a significant decrease in expression level after drought treatment (Figure 5), possibly acting as negative regulators of stress signaling. In summary, most genes in this family showed significant changes in expression levels under drought treatment, suggesting that these genes were widely involved in drought stress responses.

### 2.6. ZmTHX15 Involved in Osmotic Stress Regulation

**Figure 6:**
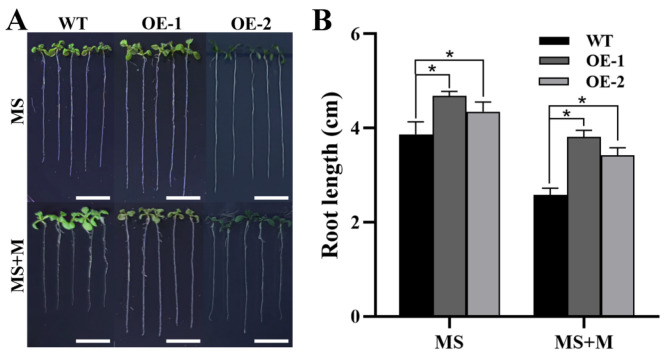
Phenotype of transgenic Arabidopsis. (**A**) The phenotypes of WT and *ZmTHX15*-overexpressing transgenic Arabidopsis line 1 and line 2 (OE-1, OE-2) treated with/without 100 mmol/L mannitol after 6 days of germination; (**B**) The root-length statistics of WT, OE-1, and OE-2 treated with/without 100 mmol/L mannitol after 6 days of germination. Data represent the mean ± SD of three biological repeats, and asterisks indicate significant differences among groups (*p* < 0.05). Bar, 1 cm.

To verify the role of *ZmTHXs* under drought stress, we constructed *ZmTHX15*-overexpressing transgenic Arabidopsis. To investigate the growth of *ZmTHX15*-overexpressing transgenic plants under osmotic stress, we treated them with mannitol. The results showed that, whether grown on regular 1/2 MS medium or medium containing 100 mM mannitol, longer root lengths were exhibited in the *ZmTHX15*-overexpressing plants compared to the WT (Figure 6A). Root length statistics of seedlings grown for 6 days revealed that, regardless of the control or mannitol treatment, the root length of *ZmTHX15*-overexpressing plants was significantly longer than that of the WT (Figure 6B). The root length of WT under mannitol treatment was 66% of the control, while the root length of transgenic plants under mannitol treatment was 82% of the control, indicating that *ZmTHX15*-overexpressing plants were insensitive to mannitol treatment (Figure 6B). The results suggest that *ZmTHX15* may play an important role in the regulation of osmotic stress.

### 2.7. ZmTHX15-Overexpressing Arabidopsis Is More Drought Tolerant than WT

**Figure 7:**
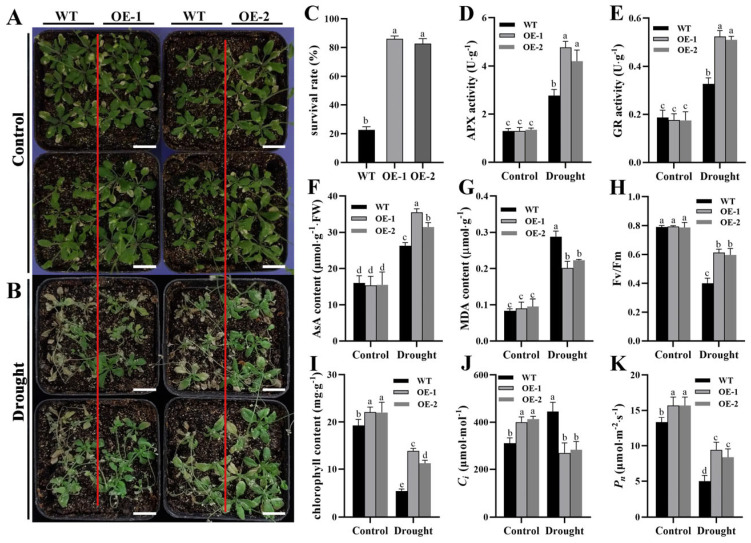
*ZmTHX15*-overexpressing *Arabidopsis* is more drought tolerant than WT. (**A**) Phenotype of WT, OE-1, and OE-2 plants transplanted for 10 days under normal watering; (**B**) Phenotype of WT, OE-1, and OE-2 plants after 10 days of drought treatment; (**C**) Plant survival rate after drought treatment; (**D**) Ascorbate peroxidase activity; (**E**) glutathione reductase activity; (**F**) ascorbic acid content; (**G**) malondialdehyde content; (**H**) chlorophyll fluorescence index; (**I**) chlorophyll content; (**J**) intercellular CO_2_ concentration; (**K**) net photosynthetic rate. The data represent the mean ± SD of three biological repeats, and the letters represent significant differences among groups (*p* < 0.05). Bar, 1 cm.

To determine whether the *ZmTHX15*-overexpressing Arabidopsis are more drought-tolerant than WT, we subjected both WT and *ZmTHX15*-overexpressing Arabidopsis plants to soil drought treatment. After 10 days of drought treatment, the WT plants showed obvious wilting, while most of the *ZmTHX15*-overexpressing plants were still able to grow normally (Figure 7A,B). Survival rate statistics indicated that after 10 days of drought treatment, the survival rate of WT plants was only about 22%, while the survival rate of *ZmTHX15*-overexpressing plants was as high as 87%, significantly higher than that of the WT (Figure 7C).

Aseorbate peroxidase (APX) and glutathione reductase (GR) are key enzymes in plants responsible for scavenging excess H_2_O_2_ and mitigating stress-induced damage. Under drought stress, plants with strong drought tolerance exhibit higher APX and GR activity compared to drought-sensitive plants. Ascorbic acid (AsA) is a non-enzymatic free radical scavenger in plants that effectively eliminates ROS generated by stress, thereby alleviating oxidative damage in plants. Based on this, we measured the activity of APX and GR, as well as the AsA content, in both WT and *ZmTHX15*-overexpressing plants before and after drought treatment. The results showed that before drought treatment, there were no significant differences in APX, GR, and AsA levels between WT and *ZmTHX15*-overexpressing plants. However, after drought treatment, the *ZmTHX15*-overexpressing plants exhibited higher APX and GR enzyme activities and accumulated more AsA (Figure 7D–F).

To investigate the oxidative damage experienced by *ZmTHX15*-overexpressing plants under drought stress, we measured the MDA content. The results indicated that the MDA content accumulated in the *ZmTHX15*-overexpressing Arabidopsis was lower than that in the WT, suggesting that the *ZmTHX15*-overexpressing plants experienced less oxidative damage under drought stress compared to the WT (Figure 7G).

To explore whether there are differences in photosynthetic efficiency between the *ZmTHX15*-overexpressing and WT plants before and after drought, we measured the photosynthetic capacity of both groups. Chlorophyll fluorescence measurements showed that the fluorescence index decreased in both WT and *ZmTHX15*-overexpressing plants after drought treatment, but the decrease was less pronounced in the *ZmTHX15*-overexpressing plants, and the fluorescence index was significantly higher than that of the WT (Figure 7H). Chlorophyll content measurements indicated that the chlorophyll content in the leaves of *ZmTHX15*-overexpressing plants was higher than that of WT after drought treatment (Figure 7I), consistent with the chlorophyll fluorescence index results.

When measuring photosynthetic rate and intercellular CO_2_ concentration, we found that before drought treatment, the CO_2_ concentration in the *ZmTHX15*-overexpressing plants was slightly higher than in the WT. After drought treatment, the situation was reversed: the intercellular CO_2_ concentration in the *ZmTHX15*-overexpressing plants decreased, while it significantly increased in the WT (Figure 7J), indicating excessive CO_2_ accumulation and a significant reduction in photosynthetic efficiency in WT. The results suggest that under drought stress, the *ZmTHX15*-overexpressing plants can continue to utilize intercellular CO_2_ for photosynthesis.

The net photosynthetic rate measurements showed that both WT and *ZmTHX15*-overexpressing plants experienced a decline in photosynthetic rate after drought treatment, but the decline was less severe in the *ZmTHX15*-overexpressing plants, and the photosynthetic rate remained significantly higher than that of WT (Figure 7K). These results indicate that *ZmTHX15*-overexpressing plants can better utilize intercellular CO_2_ under drought conditions, maintaining higher photosynthetic efficiency, which supports the normal growth under drought stress.

In summary, through experimental analysis, we found that *ZmTHX15*-overexpressing Arabidopsis exhibit stronger drought tolerance. Under drought conditions, the *ZmTHX15*-overexpressing plants suffered less secondary oxidative damage compared to the WT. The osmotic substances and antioxidants in the *ZmTHX15*-overexpressing plants worked together under drought stress to mitigate the damage caused by the stress, thereby maintaining a higher photosynthetic rate.

## 3. Discussion

In recent years, the frequent occurrence of high temperatures, low rainfall, and drought has led to significant crop yield reductions worldwide. Therefore, understanding the mechanisms of plant drought stress responses has become a major focus in the field of stress research [38]. Studies have shown that osmotic regulation, antioxidant defense systems, transcription factor regulation, and signaling pathways such as ABA play crucial roles in drought stress responses [39,40,41].

Maize, as one of the three major cereal crops, is increasingly in demand due to the growing focus on maize production and development. Therefore, research aimed at identifying key transcription factors based on maize genomic information databases is of significant scientific and practical value for understanding maize genomic information and improving maize quality.

Transcription factors are abundantly present in plants, with 56 transcription factor families and a total of 2298 members identified in maize [42]. Research has shown that the Arabidopsis genome contains more than 1500 transcription factors, accounting for over 5% of the genome [43]. Since transcription factors exert their regulatory functions by interacting with specific DNA sequences, understanding the sequences they recognize is crucial for comprehending the functions and regulatory mechanisms.

There are various transcription factor families in higher plants, most of which have been proven to be associated with plant stress responses and quality trait improvement. Transcription factors that regulate responses to drought, cold, and salinity stress have also been cloned [44,45,46]. Research on the trihelix transcription factor family in plants is an emerging field. This family has gained significant attention due to its extensive involvement in plant growth, development, and responses to various biotic and abiotic stresses. Previous studies have analyzed the trihelix transcription factor families in many plants, such as rice, wheat, maize, alfalfa, tomato, sesame, quinoa, buckwheat, and sorghum [47,48,49,50,51,52,53,54,55]. However, systematic studies on the evolution and protein structure of the maize trihelix family have not yet been fully conducted. Therefore, this study utilized the latest databases to perform a preliminary bioinformatics analysis of the maize trihelix transcription factor family. Compared to previous analyses of the trihelix transcription factor families in plants like Arabidopsis and rice, this study identified a larger number of gene family members and included additional data analysis at the gene structure and protein levels. This work contributes to the further understanding of the transcription factor family information in maize.

In this study, we conducted a phylogenetic analysis of maize trihelix transcription factor family members using bioinformatics methods. The results revealed that 46 trihelix transcription factors were identified in maize, and the family was divided into five subfamilies: GT-1, GT-2, GTγ, SIP1, and SH4, with each subfamily containing a different number of members. The GTγ subfamily has the fewest members, while the SIP1 subfamily has the most. The family members are unevenly distributed across the 10 chromosomes, and some genes exhibit gene clustering on the chromosomes. The family contains a variety of conserved motifs, and the differences in these motifs suggest the diversity of gene functions. Members of the same subfamily have similar structural characteristics. These results indicate that the maize genome may have undergone changes during evolutionary development. By predicting the biological functions of the trihelix family, the basic structure and functions of the family members were predicted, providing an important theoretical basis for future research on the role of monocot gene families in quality improvement and stress response.

## 4. Materials and Methods

### 4.1. Identification and Physicochemical Properties of ZmTHXs

The maize genome sequence, CDS sequence, genome annotation files, and protein sequences were downloaded from the EnsemblPlants database. Using the MYB binding 4 domain as a basis for searches in PlantTFDB and UniProt, the obtained sequences for domains in the Pfam, SMART, and NCBI-CDD databases were validated. After removing sequences lacking relevant domains and redundancies, 46 sequences were ultimately identified. The ExPASy (https://www.expasy.org, accessed on 7 December 2024) [56] tool was used to calculate properties such as pI, MW, instability index, and average hydrophobicity of the trihelix family genes. Subcellular localization was predicted using the Plant-mPLoc 2.0 tool (http://www.csbio.sjtu.edu.cn/bioinf/plant-multi/, accessed on 7 December 2024) [57].

### 4.2. Phylogenetic Analysis of the ZmTHXs Gene Family

Multiple sequence alignments were performed using the ClustalW tool (https://www.genome.jp/tools-bin/clustalw, accessed on 7 December 2024) [58]. The phylogenetic tree of THX protein sequences of maize, rice, and Arabidopsis was constructed using MEGAX [59] software with the Neighbor-Joining (NJ) method and 1000 bootstrap replicates. The constructed tree was imported into the iTOL v7 online software (http://itol.embl.de/, accessed on 7 December 2024) for visualization.

### 4.3. Cis-Acting Element Analysis of ZmTHXs

The 2,000 bp sequence upstream of *THX* genes start codon was extracted using TBtools [60,61]. Cis-element analysis of the promoter regions was performed using PlantCARE (http://bioinformatics.psb.ugent.be/webtools/plantcare/html/, accessed on 7 December 2024) [62]. After organizing and adjusting the analysis results, they were visualized using TBtools.

### 4.4. Chromosomal Localization and Synteny Analysis of ZmTHXs

The chromosomal location information of *ZmTHXs* genes was obtained from the maize genome annotation file, and TBtools 1.120 was used for the chromosomal localization visualization. Synteny analysis was performed using the MCScanX tool (https://github.com/wyp1125/MCScanX, accessed on 7 December 2024) [63], and the results were visualized with TBtools.

### 4.5. Differential Expression Analysis of ZmTHXs Under Drought Stress

The specific expression data of relevant genes was downloaded from the MaizeGDB-qteller database, and the gene-specific expression heat map was created using TBtools.

### 4.6. Gene Cloning

Target gene sequences were searched on the NCBI (http://www.ncbi.nlm.nih.gov, accessed on 7 December 2024) website. Primers were designed to obtain its complete cDNA using Polymerase Chain Reaction (PCR) and then cloned into the vector *pcambimGFP-1302* for sequencing. Primers are listed in Table S1.

### 4.7. Generation of Transgenic Plants

The genetic transformation of *Arabidopsis thaliana* was based on the *Agrobacterium*-mediated floral dip method [64]. After the T_1_ transgenic plants were obtained, we identified the positive plants by PCR using primers: a 35S sequence primer as the forward primer and a gene-specific primer as the reverse primer. A minimum of 12 positive transgenic lines were identified [65]. Primers are listed in Table S1.

### 4.8. Mannitol Treatment of Transgenic Arabidopsis

Sow the sterilized WT and transgenic Arabidopsis seeds separately on 1/2 MS medium containing 100 mmoL mannitol and 1/2 MS medium without mannitol. Vernalize them at 4 °C for 3 days, then transfer them to an incubator set at 22 °C with a 16/8 h light/dark cycle. Observe their phenotype daily and measure their root length using Image J (https://imagej.net/ij/, accessed on 7 December 2024) during this period [66].

### 4.9. Measurement of Chlorophyll Content

Take 0.2 g of fresh leaves and place them in a mortar, add 95% ethanol, and grind thoroughly. Let it stand for 5 min and then transfer the grinding solution to a 25 mL centrifuge tube. Centrifuge at 4000 rpm for 10 min and take the supernatant as the extract. Using ethanol as the control, measure the optical density (OD) values at wavelengths of 649 nm and 665 nm. Calculate the chlorophyll content using the following formulas:

C_a_ = 13.95A_665_ − 6.88A_649_, C_b_ = 24.96A_649_ − 7.32A_665_ (C_a_, C_b_: mg/L)

Chlorophyll content (mg/g) = (C × V)/W_f_

C = C_a_ + C_b,_

V is the volume of the extract, and W_f_ is the fresh weight of the tissue.

### 4.10. Measurement of Photosynthetic Rate (Pn) and Intercellular CO_2_ Concentration

On a clear morning, use the LI-6400XT photosynthesis system with a red/blue light source to measure the net photosynthetic rate (Pn, μmol·m^−2^·s^−1^) and intercellular CO_2_ concentration (Ci, μmol/mol) of the leaves.

### 4.11. Chlorophyll Fluorescence Measurement

Use the FluorCam 800MF chlorophyll fluorescence imaging system from Czech PSI to detect the Fv/Fm of the leaves. Open the FluorCam 800MF software on the computer, adjust the focus in the live window, select “Flashes” for the “light sources” parameter, set “act1” and “act2” to 0, and “super” to 24. Adjust the EI. Shutter to “10” and the “sensitivity” to “40”. Click “use”. After selecting the machine’s preset program, you can start the measurement. Record and save the results after the measurement is completed.

### 4.12. APX and GR Enzyme Activity

Weigh 0.1 g of Arabidopsis leaves for each sample. Use the APX Activity Assay Kit (Catalog No.: BC0220) and the GR Activity Assay Kit (Catalog No.: BC1160) from Solarbio to measure APX and GR activities, respectively. Follow the instructions in the manual for the detection steps. Perform three biological replicates for each group. Enzyme activity is expressed in units (U).

### 4.13. Determination of AsA and MDA Content

Weigh 0.1 g of Arabidopsis leaves for each sample. Use the AsA Content Assay Kit (Catalog No.: BC1230) and the MDA Content Assay Kit (Catalog No.: BC0020) from Solarbio for detection, following the steps in the manual. Perform three biological replicates for each group. AsA content is expressed in μmol·g-1, and MDA content is expressed in mmol·g^−1^.

## Figures and Tables

**Table 1:** Physicochemical properties of ZmTHXs identified in maize.

Genes	Gene ID	Amino Acids	MW (KDa)	pI	Instability Index	GRAVY	Subcellular Localization
*ZmTHX01*	Zm00001eb000880	809	84,901.28	6.55	69.6	−0.736	Nucleus
*ZmTHX02*	Zm00001eb013590	377	40,843.3	10.12	57.94	−0.649	Nucleus
*ZmTHX03*	Zm00001eb023870	321	34,106.44	9.41	74.38	−0.567	Nucleus
*ZmTHX04*	Zm00001eb033570	319	35,873.99	5.6	75.37	−0.966	Nucleus
*ZmTHX05*	Zm00001eb043580	669	70,672.04	9.22	63.48	−0.734	Nucleus
*ZmTHX06*	Zm00001eb065500	204	22,859.11	9.38	41.64	−0.412	Nucleus
*ZmTHX07*	Zm00001eb066770	381	40,870.93	9.38	80.33	−0.64	Nucleus
*ZmTHX08*	Zm00001eb071780	273	32,311.31	8.37	62.18	−1.259	Nucleus
*ZmTHX09*	Zm00001eb075080	208	22,949.16	11.09	96.5	−0.682	Nucleus
*ZmTHX10*	Zm00001eb075250	774	83,274.2	5.9	73.29	−0.882	Nucleus
*ZmTHX11*	Zm00001eb081720	385	40,978.96	6.68	64.43	−0.783	Nucleus
*ZmTHX12*	Zm00001eb084300	354	37,623.59	8.08	41.35	−0.716	Nucleus
*ZmTHX13*	Zm00001eb084860	348	37,680.89	8.69	52.69	−0.773	Nucleus
*ZmTHX14*	Zm00001eb092040	436	49,833.69	6.32	53.26	−1.023	Nucleus
*ZmTHX15*	Zm00001eb104640	357	38,923.08	8.23	32.16	−0.642	Nucleus
*ZmTHX16*	Zm00001eb129190	78	88,55.39	10.01	53.05	−0.553	Nucleus
*ZmTHX17*	Zm00001eb130740	537	57,507.76	7.1	63.71	−0.731	Nucleus
*ZmTHX18*	Zm00001eb142600	295	32,148.04	8.6	43.47	−0.617	Nucleus
*ZmTHX19*	Zm00001eb157080	318	34,724.07	9.59	70.81	−0.75	Nucleus
*ZmTHX20*	Zm00001eb180800	341	36,829.89	5.27	61.81	−0.743	Nucleus
*ZmTHX21*	Zm00001eb181760	398	45,440.35	5.97	49.73	−0.814	Nucleus
*ZmTHX22*	Zm00001eb186200	668	71,709.6	5.78	55.45	−0.758	Nucleus
*ZmTHX23*	Zm00001eb199110	262	28,997.61	9.49	64.97	−0.834	Nucleus
*ZmTHX24*	Zm00001eb202410	426	48,757.53	6.64	55.07	−1.055	Nucleus
*ZmTHX25*	Zm00001eb209880	714	76,269.47	5.93	56.8	−0.862	Nucleus
*ZmTHX26*	Zm00001eb217780	360	39,267.53	6.2	64.99	−0.882	Nucleus
*ZmTHX27*	Zm00001eb220250	777	82,583.32	6.11	57.98	−0.827	Nucleus
*ZmTHX28*	Zm00001eb229400	476	51,138.97	4.98	59.64	−0.848	Nucleus
*ZmTHX29*	Zm00001eb233350	387	40,507.55	4.55	66.65	−0.522	Nucleus
*ZmTHX30*	Zm00001eb234620	874	96,306.71	8.96	40.31	−0.296	Chloroplast
*ZmTHX31*	Zm00001eb242030	349	37,637.71	5.05	64.55	−0.662	Nucleus
*ZmTHX32*	Zm00001eb243260	405	46,187.92	5.91	47.34	−0.896	Nucleus
*ZmTHX33*	Zm00001eb248900	672	72,812.61	5.87	51.7	−0.865	Nucleus
*ZmTHX34*	Zm00001eb295700	343	36,918.88	9.91	62.4	−0.57	Nucleus
*ZmTHX35*	Zm00001eb320470	337	36,717.5	6.94	37.51	−0.631	Nucleus
*ZmTHX36*	Zm00001eb340550	533	57,191.2	6.09	57.65	−0.727	Nucleus
*ZmTHX37*	Zm00001eb360120	315	34,672.99	9.87	70.51	−0.824	Nucleus
*ZmTHX38*	Zm00001eb361590	350	38,799.78	9.7	74.3	−0.847	Nucleus
*ZmTHX39*	Zm00001eb390910	318	33,599.95	9.17	70.04	−0.458	Nucleus
*ZmTHX40*	Zm00001eb396910	251	27,105.29	6.27	61.82	−0.65	Nucleus
*ZmTHX41*	Zm00001eb407690	446	50,509.25	6.11	46.7	−1.011	Nucleus
*ZmTHX42*	Zm00001eb421380	334	36,440.24	5.89	51.63	−0.794	Nucleus
*ZmTHX43*	Zm00001eb423330	392	42,106.22	6.61	66.72	−0.846	Nucleus
*ZmTHX44*	Zm00001eb425230	379	41,935.71	6.28	48.46	−0.727	Nucleus
*ZmTHX45*	Zm00001eb427630	616	66,455.62	9.34	71.11	−0.931	Nucleus
*ZmTHX46*	Zm00001eb427790	206	22,628.8	11.19	96.72	−0.657	Nucleus

## Data Availability

The original contributions presented in this study are included in the article/Supplementary Materials. Further inquiries can be directed to the corresponding authors.

## Supplementary Materials

The following supporting information can be downloaded at: https://www.mdpi.com/article/10.3390/ijms252413257/s1.
