# Supplementary material for: Genome-Wide Identification of the Trihelix Transcription Factor Family and Functional Analysis of ZmTHX15 in Maize
Source: Int J Mol Sci. 2024 Dec 10;25(24):13257. doi: 10.3390/ijms252413257 (PMC11675602; doi:10.3390/ijms252413257)
Supplement: Supplementary file 1 [file ijms-25-13257-s001.zip › ijms-3325482-supplementary.pdf]

**Table S1.** Primers used in the paper

| Primers | Sequence 5′-3′ | Purpose |
|---|---|---|
| ZmTHX15-F | gagaacacgggggactcttgaccatggACGGCCTCCCCGACG | Gene Cloning |
| ZmTHX15-R | gtgaaaagttcttctcctttactagtACCATTCTCGCCCTTCTTGGTAAGGG | Gene Cloning |
| Se-F | ACGCACAATCCCACTATCCTTCGC | Sequencing |
| Se-R | CGTATGTTGCATCACCTTCACCCTC | Sequencing |
